# Post-Infectious Glomerulonephritis With Crescents in an Elderly Diabetic Patient: Good Prognosis

**DOI:** 10.7759/cureus.11440

**Published:** 2020-11-11

**Authors:** Turki A Banamah, Ahmed Sameer Alzahrani, Hatim Almaghraby

**Affiliations:** 1 Nephrology and Kidney Transplantation, National Guard Health Affairs, Jeddah, SAU; 2 Medicine, College of Medicine, King Saud Bin Abdulaziz University for Health Sciences, Jeddah, SAU; 3 Pathology, King Abdullah International Medical Research Centre, National Guard Health Affairs, Jeddah, SAU

**Keywords:** post-infectious glomerulonephritis, crescents, elderly diabetic, dialysis, recovery

## Abstract

Post-infectious glomerulonephritis (PIGN) is an injury to glomerules mediated by the immune response after infection, and it is commonly seen in children. However, the elderly with immunocompromised conditions are at higher risk of developing PIGN after a recent infection. A 74-year-old female presented to the ER with a history of severe, sharp, on and off left flank pain for two days. Initial laboratory results workup were suggestive of acute kidney injury with no obvious reason. Dialysis was required as the renal function was deteriorating. Serologic test was negative for ANA (anti-neutrophil antibodies), C-ANCA (anti-neutrophil cytoplasmic antibodies), and P-ANCA (perinuclear anti-neutrophil cytoplasmic antibodies). C3 level was low, and anti-streptolysin O titer was high. Renal biopsy was performed. With reference to the clinical and histological examination, she was diagnosed with PIGN and diabetic nephropathy. After six months, the renal function was improving gradually until hemodialysis was stopped, and the Permcath^TM^ (Medtronic) was removed with a creatinine level of 120 µmol/L. The elderly diabetic female developed PIGN with crescents that required dialysis, and dialysis was stopped after six months with good prognosis. Since PIGN is a very rare entity, the suspicion of PIGN in the elderly with acute kidney injury should be raised after a history of upper respiratory tract or skin infection.

## Introduction

Post-infectious glomerulonephritis (PIGN) is an injury to the glomerules mediated by an immune response after an infection, and it is commonly seen in children after a recent history of upper respiratory tract or skin infection [1,2]. The most common organism for PIGN is group A streptococcus in children and adult. PIGN is rarely seen in the elderly; however, Staphylococcus aureus is a common the causative agent [1,3]. Moreover, PIGN can be found in the elderly and other groups of patients with immunosuppression risk factors for PIGN, including conditions such as diabetes, cancer, and alcoholism, with an incidence of 0.0001% [4,5]. Unfortunately, the diagnosis is usually delayed in the elderly due to low-clinical suspicion and the lack of specific clinical signs. Only few cases of PIGN in the elderly were reported in the literature, and only a few cases were seen with a good prognosis and complete recovery. In this case study, we present a case of an elderly diabetic female who developed PIGN with crescents after upper respiratory tract infection and stopped dialysis after six months.

## Case presentation

A 74-year-old female presented to the emergency room (ER) with a history of severe, sharp, on and off left flank pain for two days, and starting gradually lasting for four hours. The pain was not radiating without any reliving or aggravating factors and was associated with nausea without vomiting. One day before the presentation, she complained of gross hematuria and dysuria twice with decreased oral intake. She had a history of upper respiratory tract infection requiring ER visits two weeks before this presentation. She denied a history of vomiting, fever, joint pain, skin rash, renal stone disease, and change in urine habit. She had a history of type 2 diabetes for 15 years, hypertension, chronic obstructive pulmonary disease (COPD), hypothyroidism, osteoporosis, osteoarthritis, and gastroesophageal reflux disease. Surgical history was unremarkable. Her medications included atorvastatin, valsartan, indapamide, omeprazole, Amlor, salbutamol, budesonide, aspirin, metformin, levothyroxine, and montelukast. There was a negative history of allergy and smoking. On examination, she was relatively well, conscious, and alert. Vital signs were as the following pulse rate of 103 bpm, respiratory rate of 16 breaths/minute, blood pressure of 168/77 mm Hg, and oxygen saturation of 96% on room air. Chest examination revealed equal, bilateral air entry with no added sound. Cardiovascular examination was normal. The abdomen was soft, with mild tenderness over the left flank with no renal angle involvement. There was no joint tenderness, swelling, skin rash, or bipedal pitting edema.

Initial laboratory results revealed a white cell count (WBC) of 15,7000/mm^3^, hemoglobin of 10.2 g/dL, platelet count of 509/mm^3^, and C-reactive protein (CRP) of 10.2 mg. The patient had a serum urea level of 10.5 mmol/L, and creatinine was 112 µmol/L compared to her baseline creatinine of 72 µmol/L. Liver function and coagulation profile were normal. Urinalysis was positive for protein (4+), and 24-hour urine protein was 5.4 g. Ultrasound for both kidneys showed normal kidney size with no obstruction. Chest X-ray, ECG, and echocardiogram were normal. 

**Figure 1:**
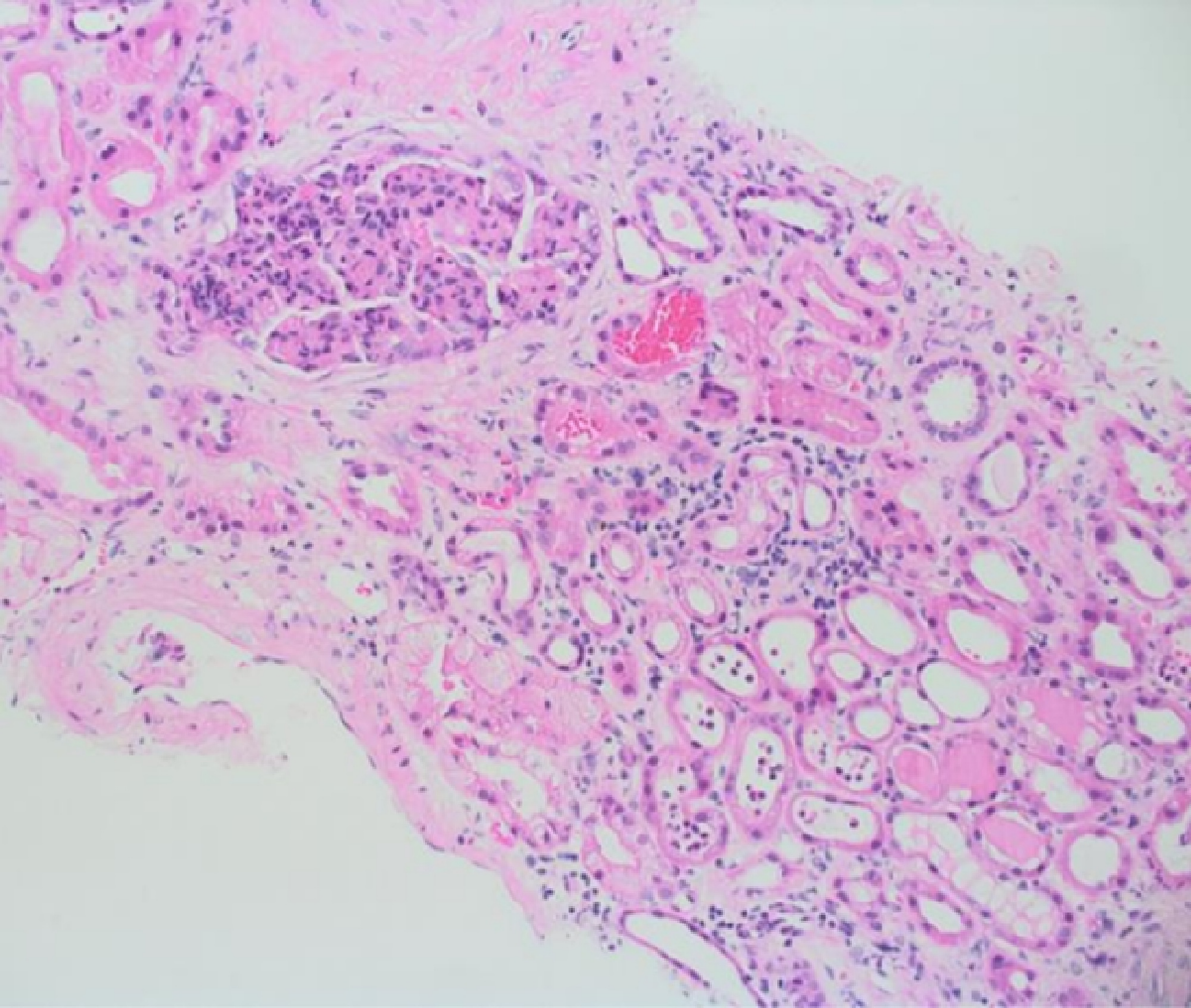
Hypercellular glomeruli showing diffuse endocapillary proliferation (arrow), H&E routine stain, x200.

As initial laboratory results workup suggested acute kidney injury with no obvious reason, she was admitted to the medical ward. Septic workup and serological tests were performed, and kidney biopsy was arranged. One day after the admission, her urine output was decreasing, and creatinine was escalating till it reached 530 within three days from admission. Dialysis was required as the renal function was deteriorating. Serologic test was negative for ANA (anti-neutrophil antibodies), C-ANCA (anti-neutrophil cytoplasmic antibodies), and P-ANCA (perinuclear anti-neutrophil cytoplasmic antibodies). C3 level was 0.16 g/L, which is low, and C4 was normal. Anti-streptolysin O titer was 890 IU /mL, which was high. Her septic workup and urine culture were negative. A renal biopsy was performed, and the glomeruli showed diffuse proliferative glomerulonephritis. Sections showed cores of renal biopsy tissue containing up to 10 glomeruli, one of which was globally sclerosed. In the remaining glomeruli, there was diffuse global obliteration of capillary loops by endocapillary hypercellularity composed of mesangial cells, endothelial cells, and neutrophil. Three glomeruli showed cellular crescents (Figure 1).

**Figure 2:**
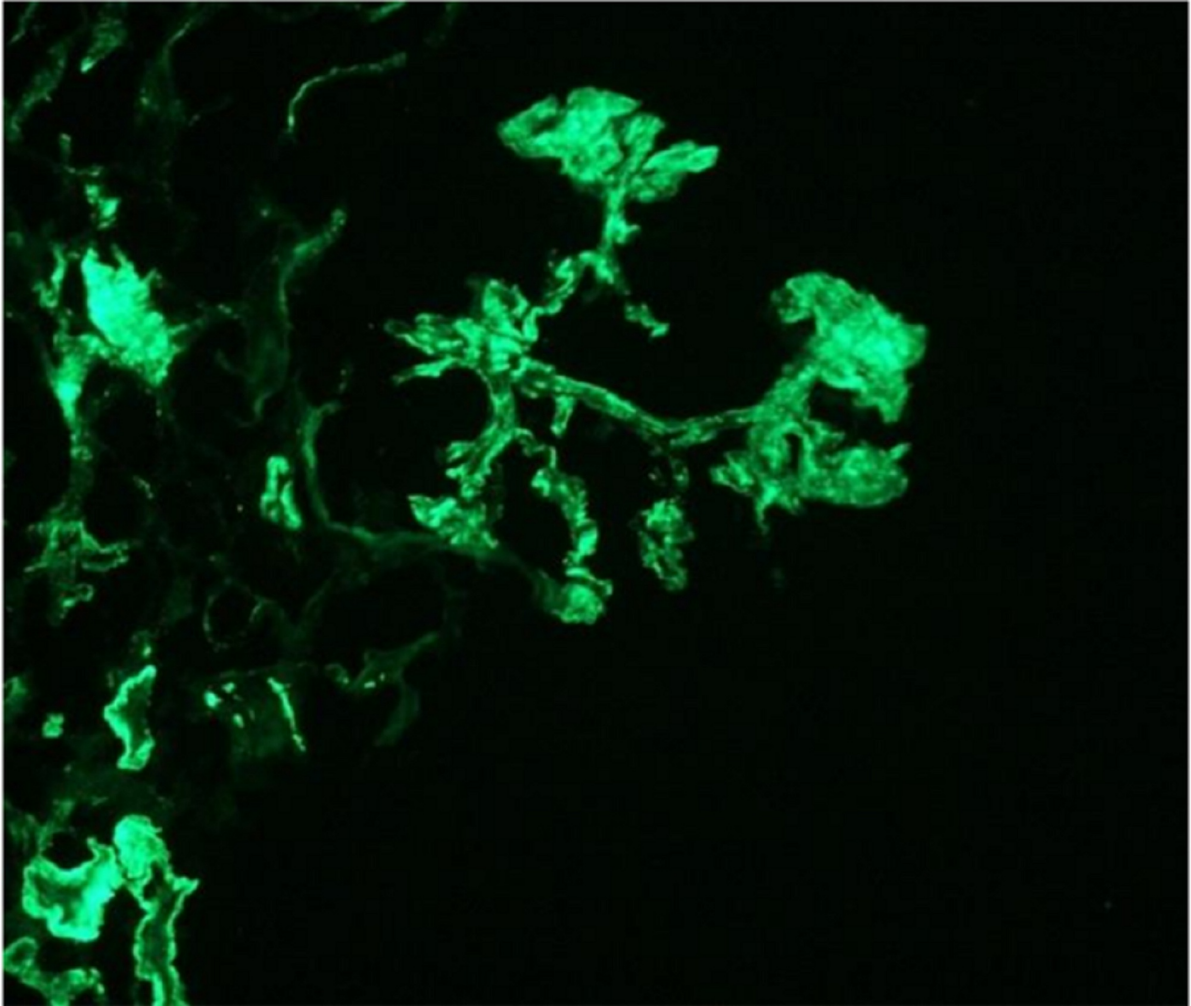
Immunofluorescence demonstrated granular capillary wall and predominantly mesangial granular positivity to anti-sera directed against C3, IgA, IgG, kappa, and lambda (x400). IG, immunoglobulin

There was moderate interstitial fibrosis, and tubular atrophy was present. Moreover, the lumen contained neutrophils, red blood cells, and granular cast. Immunofluorescence evaluation showed granular mesangial and capillary wall positivity to anti-sera directed against C3 (+4), IgG (+1), immunoglobulin (Ig) A (+2), kappa (+1), and lambda (+1) (Figure 2).

**Figure 3:**
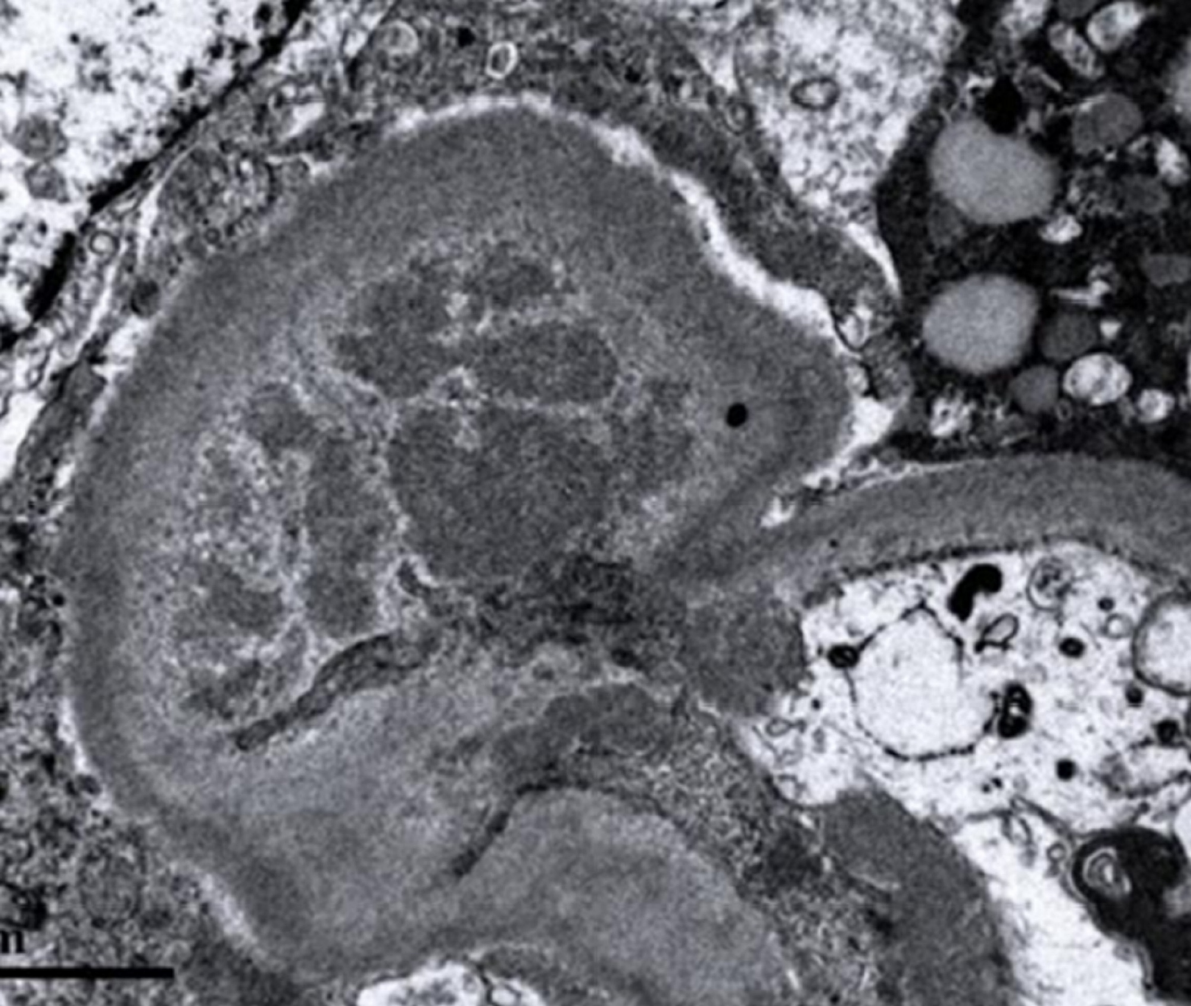
Mesangial variable-size multiple well-defined immune-type electron-dense deposits are noted, electron microscopy, x8500.

Electron microscopy revealed numerous small- and medium-size mesangial, subepithelial, and subendothelial immune-type electron-dense deposits. The glomerular basement membrane showed mild diffuse thickening consistent with the history of type II diabetes mellitus (Figure 3).

In view of the findings, the patient was diagnosed as PIGN in a background of diabetic nephropathy. 

Initially, the patient received meropenem, and pulse steroid was given for three days and then followed by oral steroid and one dose of cyclophosphamide, but she was not improving. The patient required ICU admission for three days due to hospital-acquired pneumonia and COPD exacerbation leading to type 2 repository failure. On discharge from the ICU, she was stable on antibiotics, and steroid was tapered quickly. A permanent catheter (Permcath^TM^, Medtronic, Minneapolis, MN, USA) was inserted for hemodialysis, and the patient was discharged with good status of health. After six months, the renal function was improving gradually until hemodialysis was stopped, and the Permcath was removed with a creatinine level of 120 µmol/L.

## Discussion

The prevalence of PIGN has decreased over the past three decades because of early recognition and treatment. In the elderly, immunocompromised conditions are considered risk factors to develop PIGN; the most common risk factor is diabetes followed by malignancy. Skin, lung, urinary tract, and upper respiratory tract infections are the most commonly related conditions to glomerulonephritis in the elderly [6]. The most common organisms are *Staphylococcus* and gram-negative organisms. The differential diagnosis of PIGN in the elderly is based on complement level as a typical feature of PIGN is the low level of C3. C4 level is usually normal, and ANA, C-ANCA, and P-ANCA are usually negative [4].

The most common finding of PIGN on light microscopy is diffuse endocapillary proliferation with exudates [6]. The most common staining pattern in immunofluorescence is C3 with one or more of C4, IgM, and, rarely, IgA [4].

IgA-dominant PIGN is a rare type of PIGN, and it is usually seen in the elderly with diabetes. The most common organisms for PIGN are *Staphylococcus* and gram-negative organisms. IgA PIGN patients present with acute kidney injury with severe proteinuria, and most of them have chronic renal disease [7].

Ultrastructurally, 92% of PIGN patients have subepithelial immune-type electron-dense deposits, with the presence of mesangial deposits seen in 87% of patients and subendothelial deposits detected in 66% of patients [6].

Given the rareness of the entity among the elderly, treatment is highly difficult and is on a case-by-case basis with guidelines. The mainstay of treatment is penicillin, and anti-hypertensives, diuretics, and dialysis can be added in case of circulatory overload [8]. Moreover, the response to immunosuppressive therapy is not effective compared to other types of glomerulonephritis [3].

Most of the elderly with PIGN have a poor prognosis. Nasr et al. found out that 33% and 44% had end-stage renal disease and persistent renal dysfunction, respectively, whereas only 22% recovered completely; 72 patients with PIGN showed that 16 patients required dialysis at the time of diagnosis and came off dialysis. Of these 16 patients, 5 patients achieved complete recovery, and 10 patients had persistent renal dysfunction [6].

The present case provides variation in terms of causative organisms and prognosis from usual PIGN elderly cases. The good prognosis could be attributed to the early identification and treatment; thus, a high index of suspension of PIGN should be given in multi-comorbid cases with acute kidney injury, even in the absence of a clear recent history of an upper respiratory tract or skin infection.

## Conclusions

We presented a case of an elderly diabetic female who developed PIGN with crescents. Since PIGN is a very rare entity and it has no diagnostic symptoms or signs, an early suspicion of PIGN in multi-comorbid elderly patients with acute kidney injury should be raised to provide prompt treatment and prevent further complications.
